# Crowdsourcing Diagnosis for Patients With Undiagnosed Illnesses: An Evaluation of CrowdMed

**DOI:** 10.2196/jmir.4887

**Published:** 2016-01-14

**Authors:** Ashley N.D Meyer, Christopher A Longhurst, Hardeep Singh

**Affiliations:** ^1^ Houston Veterans Affairs Center for Innovations in Quality, Effectiveness and Safety Health Services Research and Development Michael E. DeBakey Veterans Affairs Medical Center Houston, TX United States; ^2^ Section of Health Services Research Department of Medicine Baylor College of Medicine Houston, TX United States; ^3^ Department of Biomedical Informatics University of California San Diego La Jolla, CA United States

**Keywords:** crowdsourcing, diagnosis, diagnostic errors, patient safety, World Wide Web

## Abstract

**Background:**

Despite visits to multiple physicians, many patients remain undiagnosed. A new online program, CrowdMed, aims to leverage the “wisdom of the crowd” by giving patients an opportunity to submit their cases and interact with case solvers to obtain diagnostic possibilities.

**Objective:**

To describe CrowdMed and provide an independent assessment of its impact.

**Methods:**

Patients submit their cases online to CrowdMed and case solvers sign up to help diagnose patients. Case solvers attempt to solve patients’ diagnostic dilemmas and often have an interactive online discussion with patients, including an exchange of additional diagnostic details. At the end, patients receive detailed reports containing diagnostic suggestions to discuss with their physicians and fill out surveys about their outcomes. We independently analyzed data collected from cases between May 2013 and April 2015 to determine patient and case solver characteristics and case outcomes.

**Results:**

During the study period, 397 cases were completed. These patients previously visited a median of 5 physicians, incurred a median of US $10,000 in medical expenses, spent a median of 50 hours researching their illnesses online, and had symptoms for a median of 2.6 years. During this period, 357 active case solvers participated, of which 37.9% (132/348) were male and 58.3% (208/357) worked or studied in the medical industry. About half (50.9%, 202/397) of patients were likely to recommend CrowdMed to a friend, 59.6% (233/391) reported that the process gave insights that led them closer to the correct diagnoses, 57% (52/92) reported estimated decreases in medical expenses, and 38% (29/77) reported estimated improvement in school or work productivity.

**Conclusions:**

Some patients with undiagnosed illnesses reported receiving helpful guidance from crowdsourcing their diagnoses during their difficult diagnostic journeys. However, further development and use of crowdsourcing methods to facilitate diagnosis requires long-term evaluation as well as validation to account for patients’ ultimate correct diagnoses.

## Introduction

Errors of clinical diagnosis affect at least 5% of US adults every year and approximately half of these errors could result in serious harm to the patients [1]. To address the extent and severity of this problem, both systems and cognitive solutions have been proposed. However, only a few of these have been tested and only a fraction of those tested have been shown to improve diagnostic outcomes [2-4]. Patients with difficult-to-diagnose conditions often seek care from several physicians and institutions before obtaining a diagnosis. One intervention that could benefit patients is the use of second opinions [5-7], and this has been shown to catch previously missed diagnoses, at least in the realms of radiology and pathology [6]. Several formal programs currently exist to provide second opinions to patients. [7] For example, in the NIH Undiagnosed Diseases Network based at several centers across the US [8], medical experts diagnose undiagnosed individuals or those with rare diseases. The program, however, has strict eligibility requirements for patients and requires a clinician referral. Additional programs include Best Doctors’ second-opinion program that is open to employee beneficiaries only and Cleveland Clinic’s MyConsult program [5,9], both of which involve comprehensive review of patients’ medical records, but no dynamic interactions with the patients.

A recently developed software platform, CrowdMed [10], aims to overcome some limitations of the aforementioned programs, namely; strict eligibility requirements, needed referrals, and limited interaction with patients; by leveraging the “wisdom of the crowd” or crowdsourcing to help undiagnosed or misdiagnosed patients. Crowdsourcing is a “participative online activity” in which a group of individuals of varying knowledge, heterogeneity, and number comes together to solve a problem [11]. It has been used for a variety of problems in different fields ranging from simple text translation to more complicated tasks, such as solving the BP oil spill disaster in the Gulf of Mexico [12]. In medicine, it has been utilized for health and medical research, such as estimating flu prevalence [13]; for informatics solutions, including establishing problem-related medication pairs [14], and for examining specific diseases through image analysis. In the latter situation, crowdsourcing has been used to inspect blood samples to determine the presence or absence of malarial infections [15-17] and to categorize colorectal polyps [18,19] or diabetic retinopathy [20]. However, until now crowdsourcing had not been used to come up with a diagnosis from all possible diagnoses a patient might have. Of note, this platform allows laypersons without health care training or experience to participate. Although patients have been “googling for a diagnosis” for more than a decade and even using online symptom checkers [21,22], this is the first description of a crowd of people working together online towards a more accurate diagnosis. We conducted an independent evaluation of this untested approach to determine whether this could be beneficial to patient care.

## Methods

### A Description of CrowdMed

**Figure 1:**
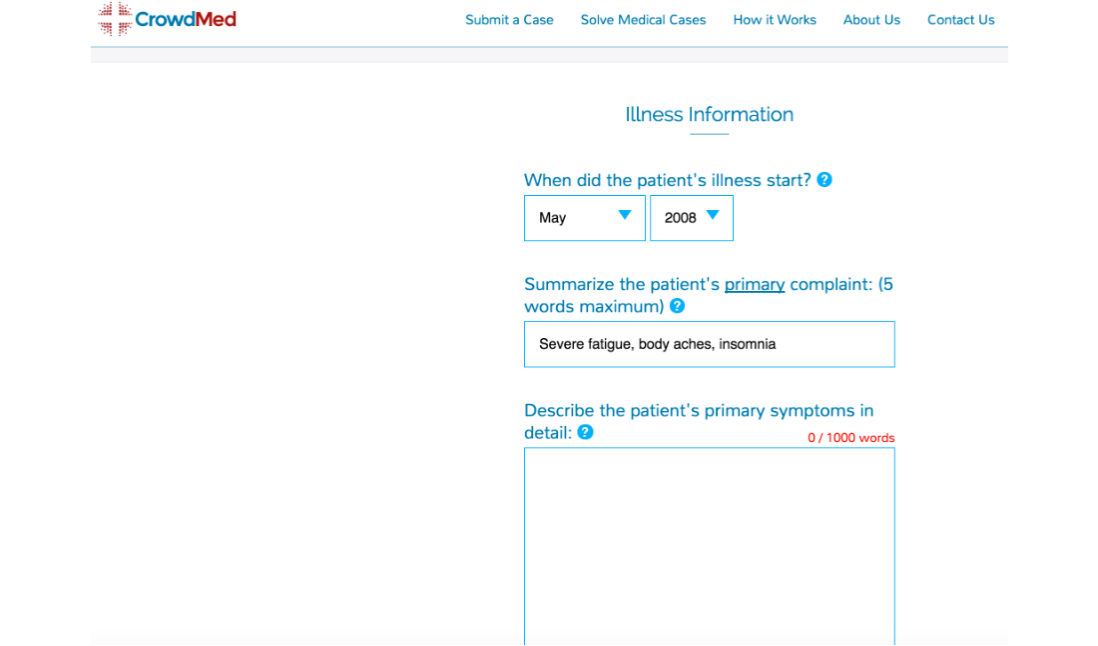
Screenshot of case submission.

For a small fee, the CrowdMed website allows undiagnosed patients to submit their clinical information and obtain potential diagnoses expeditiously. Patients anonymously answer a comprehensive set of medical questions and upload relevant test results and images related to their cases (Figure 1).

**Figure 2:**
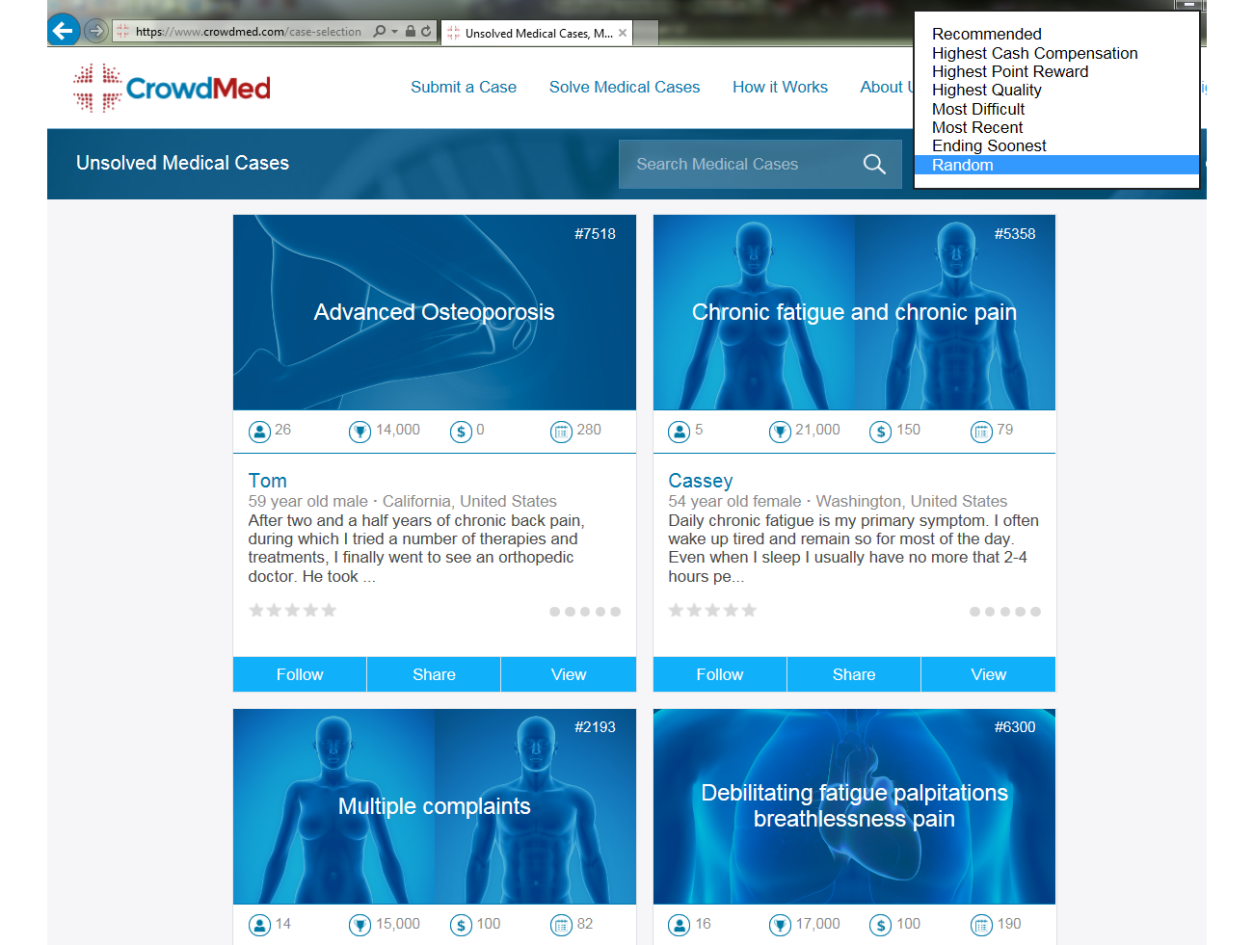
Screenshot of case selection for solvers (names are fictitious).

Patients also decide how long they want their cases open and whether they wish to compensate the case solvers. Anyone (including nonmedical persons) can sign up to be a case solver and select cases they think they can help solve (Figure 2).

While the cases are open, patients and case solvers can discuss details online about potential diagnoses, further work-up that should be done, and newly obtained test results and/or appointments completed with the patients’ physicians. Thus, case details can unfold online while the case is still open. All diagnostic suggestions and all case discussions are available to all case solvers as they are suggested and discussed throughout the open period. This enables the entire group of case solvers to work in concert to solve each case.

When a patient’s case is closed, the patient receives a detailed report containing the entire list of diagnostic suggestions made by the case solvers and suggested next steps, so that they can discuss them with their physicians. Diagnoses are ranked in decreasing order of “relative popularity.” The relative popularity of diagnoses is determined by case solvers’ “bets” on each diagnosis in terms of their beliefs that the diagnosis is the most specific, accurate, root cause of the symptoms presented. CrowdMed takes these bets and assigns points to each diagnosis using a prediction market algorithm, thereby determining the “relative popularity” of each diagnosis suggested. Finally, patients are provided with case solvers’ reasoning for choosing particular diagnoses. Patients choose which case solver(s) to compensate based on whose answers they found helpful. If the patient decides to reward multiple solvers, they also decide how to divvy up the compensation. Afterward, patients are invited to fill out surveys about their outcomes.

### Independent Evaluation

We independently analyzed all CrowdMed data collected from May 2013 to April 2015. Specifically, we analyzed data on patients’ demographic and case characteristics; case solvers’ demographic and performance characteristics; and preliminary case outcomes. Outcomes included whether patients would recommend CrowdMed, if the program provided insights leading them closer to correct diagnoses, and estimated improvements in patients’ productivity and medical expenses. Data were summarized using descriptive statistics and independent samples *t* tests using IBM SPSS Statistics 22.

## Results

### Patients and Cases

During the study period, 397 cases were completed (350 from the United States). Patients’ self-reported mean (SD) age was 47.8 (18.8) years (age range 2-90) and 182 were males (45.8%).

Before case submission, patients reported visiting a median of 5 physicians (interquartile range [IQR] 3-10; range 0-99), incurred a median of US $10,000 in medical expenses (IQR US $2500-US $50,000; range US $0-US $5,000,000) including payments by both patients and payers, spent a median of 50 hours (IQR 15-150; range 0-12,000) researching their illnesses online, and had symptoms for a median of 2.6 years (IQR 1.1-6.9; range 0.0-70.6). Online case activity lasted a median of 60 days (IQR 30-90; range 2-150) and case solvers were offered a median of US $100 in compensation (IQR US $0-US $200; range US $0-US $2700) for diagnostic suggestions. A total of 59.7% (237/397) of the cases were compensated with a median compensation of US $200 (IQR US $100-US $300; range US $15-US $2700).

### Case Solvers

During the study period, CrowdMed had 357 active case solvers; of which 37.9% (132/348) were male, 76.7% (264/344) were from the US, and 58.3% (208/357) worked or studied in the medical industry; including 36 physicians and 56 medical students. Mean (SD) age was 39.6 (13.8) years (range 17-77 years).

Solvers participated in a median of 3 cases (IQR 1.0-12.8; range 0-415), earned a median of US $0 (IQR US $0-US $1.18; range US $0-US $3952) and a mean (SD) of US $93.97 (US $364.72; the majority earned US $0). Median solver rating was 3 (out of 10; IQR 3-6; range 1-10) and significantly higher (*P*=.006) for medical industry-based solvers (mean [SD] 4.8 [2.5]; range 1-10) than for others (mean [SD] 4.1 [2.2]; range 1-10).

### Outcomes

At completion, 50.9% (202/397) of patients were likely to recommend CrowdMed to a friend, 59.6% (233/391) reported that the process gave insights leading them closer to correct diagnoses, 57% (52/92) reported estimated decreases in medical expenses, and 38% (29/77) reported estimated improvements in school or work productivity (Table 1).

**Table 1:** Case outcomes as assessed in a postcase survey.

Case outcomes		n (%)
**On a scale of 1-5, How likely are you to recommend CrowdMed to a friend (with 5 being most likely)?** **(391/397 surveyed answered; 98.5% response rate)**		
	1	39 (10.0)
	2	43 (11.0)
	3	107 (27.4)
	4	76 (19.4)
	5	126 (32.2)
**Did CrowdMed Medical Detective community provide insights that lead you closer to a correct diagnosis or cure?** **(391/397 surveyed answered; 98.5% response rate)**		
	No	158 (40.4)
	Yes	233 (59.6)
**How much do you estimate that your CrowdMed results will reduce the cost of your medical case going forward?** **(92/147 surveyed answered; 62.6% response rate)** ^a^		
	1-20%	25 (27.2)
	21-50%	15 (16.3)
	51-80%	10 (10.9)
	>80%	2 (2.2)
	Not at all	40 (43.5)
**How much lost work or school productivity do you estimate that your CrowdMed results will help you regain going forward?** **(77/147 surveyed answered; 52.4% response rate)** ^a^		
	1-20%	12 (15.6)
	21-50%	8 (10.4)
	51-80%	7 (9.1)
	81-99%	1 (1.3)
	All	1 (1.3)
	None	48 (62.3)

^a^These questions were added to the postcase survey later.

Patients reporting helpful insights from CrowdMed saw fewer doctors (mean [SD] 7.2 [7.3]; range 0-99) before participating than those who did not report receiving helpful insights (mean [SD] 9.2 [10.7]; range 0-50), *P*=.047. The 14 most common diagnoses suggested as the most popular diagnosis for a case are presented in Table 2.

**Table 2:** The 14 most common diagnoses suggested as the most popular diagnosis across 397 cases.

Diagnosis	n (%)
Lyme disease	8 (2.0)
Dysautonomia	7 (1.8)
Chronic fatigue syndrome	6 (1.5)
Irritable bowel syndrome	6 (1.5)
Mast cell activation disorder	6 (1.5)
Postural orthostatic tachycardia syndrome	5 (1.3)
Ehlers-Danlos syndrome	4 (1.0)
Sjögren’s syndrome	4 (1.0)
Abdominal cutaneous nerve entrapment syndrome	3 (0.8)
Gastroesophageal reflux disease	3 (0.8)
Hypothyroidism	3 (0.8)
Multiple sclerosis	3 (0.8)
Myasthenia gravis	3 (0.8)

In addition, some patients informally reported to CrowdMed that the program helped them find diagnoses that their physicians previously were unable to determine, including Sjögren’s syndrome and chorda tympani dysfunction.

## Discussion

### Main Findings

Our independent evaluation suggests that at least some patients with undiagnosed illnesses reported receiving helpful guidance from crowdsourcing their diagnoses during their difficult diagnostic journeys. Several of the conditions most commonly suggested by case solvers are conditions well known to represent diagnostic challenges. The crowdsourcing strategy enabled dynamic interaction between patients and case solvers as more case details unfolded over time.

Novel approaches are needed to help patients who experience difficulties in obtaining a correct and timely diagnosis. In that regard, advantages of using “wisdom of the crowd” could include low cost, increased program accessibility for patients, and relatively quick opinions. Although the data we obtained were useful for understanding this program, there were several limitations of our study. The postparticipation survey was rather limited in scope as it was designed for business purposes and not for research. In addition, there was no way to verify patient-reported data and some patient-reported data might be outside of realistic boundaries (eg, 1 patient reported spending 12,000 hours researching illnesses online). Furthermore, downstream outcomes of patients were not systematically collected, so it is not known what their eventual diagnoses were or if the program identified them accurately. Further development and use of crowdsourcing methods to facilitate diagnosis requires long-term evaluation as well as validation to account for patients’ ultimate correct diagnoses.

Although crowdsourcing appears to have potential, it is important to identify factors that lead to successful crowdsourcing to improve the process and help improve patient care. Multidisciplinary research is needed to gain both technical and nontechnical insights into how this can be done. For example, previous researchers have identified the importance of both finding crowd members with the appropriate skills to the relevant problem and providing adequate motivation to the crowd for the successful use of crowdsourcing for problem solving [23]. Finally, the potential legal ramifications of giving individuals without medical degrees (who make up a substantial portion of the case solvers) the ability to render diagnostic opinions would need to be considered [24].

### Conclusions

In conclusion, our independent evaluation suggests that some patients with undiagnosed illnesses report receiving helpful guidance from crowdsourcing their diagnosis. Further development and use of crowdsourcing methods to facilitate diagnosis require multidisciplinary research and long-term evaluation that includes validation to account for patients’ ultimate correct diagnoses.
